# A study protocol for quantitative targeted absolute proteomics (QTAP) by LC-MS/MS: application for inter-strain differences in protein expression levels of transporters, receptors, claudin-5, and marker proteins at the blood–brain barrier in ddY, FVB, and C57BL/6J mice

**DOI:** 10.1186/2045-8118-10-21

**Published:** 2013-06-08

**Authors:** Yasuo Uchida, Masanori Tachikawa, Wataru Obuchi, Yutaro Hoshi, Yusuke Tomioka, Sumio Ohtsuki, Tetsuya Terasaki

**Affiliations:** 1 Division of Membrane Transport and Drug Targeting, Graduate School of Pharmaceutical Sciences, Tohoku University, 6-3 Aoba, Aramaki, Aoba-ku, Sendai, 980-8578, Japan; 2 Department of Pharmaceutical Microbiology, Faculty of Life Sciences, Kumamoto University, 5-1 Oe-honmachi, Kumamoto, 862-0973, Japan

**Keywords:** Quantitative targeted absolute proteomics (QTAP), Pharmacoproteomics (PPx), Absolute expression level, *In silico* peptide selection criteria, LC-MS/MS, Blood–brain barrier, Strain difference, Transporter, Receptor, Tight junction protein

## Abstract

Proteomics has opened a new horizon in biological sciences. Global proteomic analysis is a promising technology for the discovery of thousands of proteins, post-translational modifications, polymorphisms, and molecular interactions in a variety of biological systems. The activities and roles of the identified proteins must also be elucidated, but this is complicated by the inability of conventional proteomic methods to yield quantitative information for protein expression. Thus, a variety of biological systems remain “black boxes”. Quantitative targeted absolute proteomics (QTAP) enables the determination of absolute expression levels (mol) of any target protein, including low-abundance functional proteins, such as transporters and receptors. Therefore, QTAP will be useful for understanding the activities and roles of individual proteins and their differences, including normal/disease, human/animal, or *in vitro*/*in vivo*. Here, we describe the study protocols and precautions for QTAP experiments including *in silico* target peptide selection, determination of peptide concentration by amino acid analysis, setup of selected/multiple reaction monitoring (SRM/MRM) analysis in liquid chromatography–tandem mass spectrometry, preparation of protein samples (brain capillaries and plasma membrane fractions) followed by the preparation of peptide samples, simultaneous absolute quantification of target proteins by SRM/MRM analysis, data analysis, and troubleshooting. An application of QTAP in biological sciences was introduced that utilizes data from inter-strain differences in the protein expression levels of transporters, receptors, tight junction proteins and marker proteins at the blood–brain barrier in ddY, FVB, and C57BL/6J mice. Among 18 molecules, 13 (abcb1a/mdr1a/P-gp, abcc4/mrp4, abcg2/bcrp, slc2a1/glut1, slc7a5/lat1, slc16a1/mct1, slc22a8/oat3, insr, lrp1, tfr1, claudin-5, Na^+^/K^+^-ATPase, and γ-gtp) were detected in the isolated brain capillaries, and their protein expression levels were within a range of 0.637-101 fmol/μg protein. The largest difference in the levels between the three strains was 2.2-fold for 13 molecules, although bcrp and mct1 displayed statistically significant differences between C57BL/6J and the other strain(s). Highly sensitive simultaneous absolute quantification achieved by QTAP will increase the usefulness of proteomics in biological sciences and is expected to advance the new research field of pharmacoproteomics (PPx).

## Background

**Figure 1:**
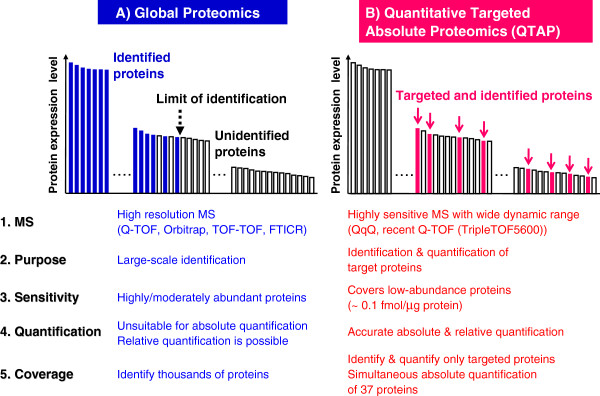
Comparison of global proteomics (A) and quantitative targeted absolute proteomics (QTAP) (B).

Proteomics by name is almost 20 years old and has rapidly grown into one of the most active research areas in biological sciences. Proteomics has had tremendous impacts on a variety of biological fields. Mass spectrometry (MS)-based protein identification is now widely adopted, and recent advances in MS and global proteomics (Figure 1), including protein sequence databases, have enabled the identification of hundreds to thousands of proteins in biological materials in a single analysis [1-3]. However, the proteome coverage attainable with available global proteomic approaches remains insufficient. Highly abundant proteins are easy to identify, but low-abundance proteins are difficult to detect due to high background noise when analyzing complex samples (Figure 1). Physiologically relevant molecules with low protein expression levels, such as transporters and receptors, are not readily identified by current global proteomic technologies. Improvements in fractionation, purification, and separation techniques in sample preparation and liquid chromatography (LC) and increased resolution and sensitivity of MS devices are required but remain challenging. Furthermore, the activities and roles of individual proteins must be elucidated, but this is hampered by the lack of quantitative information for protein expression in global proteomics.

Quantitative targeted absolute proteomics (QTAP) represents a new generation of proteomic methods that has permitted the determination of absolute protein expression levels (mol) of target proteins in biological materials by liquid chromatography-linked tandem mass spectrometry (LC-MS/MS) (Figure 1) [4]. Selected/multiple reaction monitoring (SRM/MRM) in the MS/MS devices is an essential analytical mode in QTAP that allows the target peptides of target proteins to be distinguished in significantly complex samples and provides high selectivity and a high signal-to-noise ratio (Figure 1). Because triple quadrupole (QqQ) MS has excellent sensitivity and a wide dynamic range, SRM/MRM analysis with QqQ MS permits highly sensitive quantification of target proteins and is the most widely used method for QTAP (Figure 1). For these reasons, QTAP is useful to understand the activities of functional proteins, including low-abundance molecules.

The selection of target peptides for target proteins is a critical but rate-limiting step to achieve highly sensitive and reliable protein quantification in QTAP. To solve this problem, we have established a method to design appropriate target peptides *in silico* from sequence information in protein databases (Table 1) [4]. Hence, we have succeeded in quickly developing LC-MS/MS quantification methods for several proteins. We have quantified more than 100 molecules, including transporters and receptors in the human blood–brain barrier (BBB), and have elucidated inter-species differences in protein expression levels between humans, the cynomolgus monkey, and the ddY mouse [4-6]. Furthermore, the quantitative protein expression profiles of many molecules have been applied to validate the use of the human BBB model cell line (hCMEC/D3) *in vitro* by comparison with *in vivo* human BBB cells [7].

**Table 1:** ***In silico *peptide selection criteria**

		
**Necessary conditions**	1.	The peptide is theoretically obtained by a protease, i.e., trypsin digestion of the target protein. An arginine or lysine residue occurs prior to the site of cleavage and at the C-terminus of the peptide if trypsin is used.
	2.	The amino acid sequence of the peptide is unique for a target protein in the peptide library that is theoretically obtained by protease digestion of all the proteins that are registered in protein databases.
	3.	A length of 6 to 16 amino acids (8 to 10 amino acids is preferable) for detection by QqQ MS.
	4.	NO methionine or cysteine residues are included.
	5.	NO posttranslational modification and NO single nucleotide polymorphisms are included for the quantification of the total level of the target protein.
	6.	NO continuous sequence of arginine or lysine residues (RR, KK, RK, KR) occurs in the digestion region for efficient digestion by trypsin.
	7.	The peptide does NOT include a proline residue at the C-terminal side of an arginine or lysine residue (RP or KP) in the digestion region for efficient digestion by trypsin.
	8.	The peptide does NOT include a transmembrane region for efficient digestion by a protease (such as trypsin).
**Sufficient conditions**	9.	The peptide does NOT include histidine residues, which reduce peptide sensitivity in the mass spectrometer.
	10.	The peptide includes a glycine or proline residue to increase peptide sensitivity in the mass spectrometer.
	11.	The LC retention time should be predicted based on the hydrophobicity of the amino acids.
	12.	A water-soluble peptide should be selected based on the hydrophobicity of the amino acids. Hydrophobic amino acids should comprise less than 40% of the peptide.

The table is taken from Kamiie et al. [4] with some modification.

Quantitative assays using antibodies, such as quantitative western blotting and ELISA, are widely used for protein quantification. However, these assays have significant disadvantages, including the lack of suitable specific antibodies for many proteins and the difficulty in obtaining these antibodies. By contrast, QTAP permits the development of appropriate LC-MS/MS-based absolute quantification methods for almost any target protein if sequence information is registered in the protein databases. The dynamic range of quantification in QTAP is significantly wider than that in antibody-based quantification. Furthermore, QTAP method development requires only 1 month, which is considerably more rapid than antibody development [8].

Another advantage of QTAP is the ability to reconstruct the *in vivo* activities of individual target molecules by integrating the molecular activities measured *in vitro* with *in vitro/in vivo* differences in protein expression levels [9]. The major limitation of *in vivo* functional analysis using imaging technologies such as positron emission tomography (PET) and single photon emission computed tomography (SPECT) is the difficulty of accurately evaluating the specific activity of target proteins because the specificities for tracers are often similar among protein molecules, including functionally unknown proteins. QTAP can solve this problem based on the *in vitro*-to-*in vivo* reconstruction theory and is expected to advance the new research field of pharmacoproteomics (PPx).

The purpose of the present manuscript is to provide detailed protocols and precautions for QTAP experiments. To demonstrate the usefulness and limitations of QTAP, an application of QTAP in biological sciences is introduced that utilizes data from inter-strain differences in the protein expression levels of transporters, receptors, tight junction proteins, and marker proteins at the blood–brain barrier (BBB) in ddY, FVB, and C57BL/6J mice.

## Methods and design

### Workflow of QTAP

**Figure 2:**
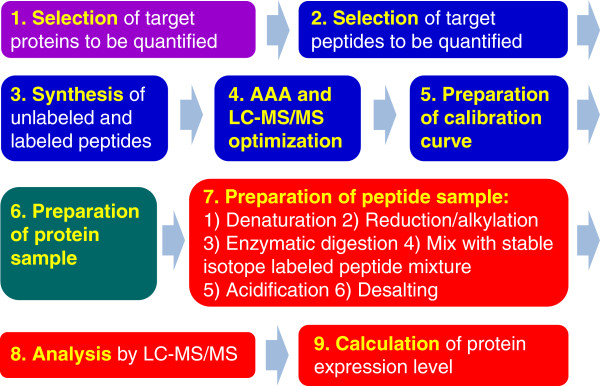
**Basic workflow of quantitative targeted absolute proteomics (QTAP).** Blue boxes (steps 2 to 5) correspond to section “Method Setup for QTAP”. The green box (step 6) corresponds to section “Preparation of protein samples”. The red boxes (steps 7 to 9) correspond to section “Absolute quantification by LC-MS/MS”. AAA, amino acid analysis.

The QTAP experiment consists of 9 steps, and the basic workflow is outlined in Figure 2. Step 1 is the selection of the target proteins to be quantified. Global proteomics, protein chip technology, mRNA analysis using PCR or DNA chip technology, and other methodologies can be used to conduct global screening of the proteins that will be targeted in QTAP.

Step 2 is the selection of the target peptide sequences for the target proteins. The target peptides are selected *in silico* based on the peptide selection criteria (Table 1). The peptide should have the following features: unique amino acid sequence, efficient protease digestion, appropriate LC retention time, and good MS sensitivity. It is important to predict the MS sensitivity of the peptide before analysis because sensitivity can vary by 1 million-fold, depending on the amino acid sequences of the peptides [10].

Step 3 is the synthesis of a stable-isotope labeled peptide that will be used as an internal standard (IS) and an unlabeled 95% pure peptide that will be used to establish a calibration curve.

Step 4 is the accurate determination of the concentration of the peptide solution by quantitative amino acid analysis (AAA) and the optimization of the LC-MS/MS conditions, including SRM/MRM transitions, declustering potentials (DP), and collision energies (CE).

Step 5 is the construction of the calibration curve using a mixture of a dilution series of the unlabeled peptide and a fixed amount of the labeled peptide. The peptide mixture is injected onto the C18 column of the LC coupled with MS/MS to confirm the sensitivity and accuracy of the optimized SRM/MRM analysis and appropriate peptide separation on the column.

Step 6 is the preparation of the protein samples. QTAP is applicable to several types of protein samples, including those used in ELISA or immunoblotting. We have already applied QTAP for whole tissue lysates of human tissues; monkey and mouse brain capillaries; whole cell lysates of hCMEC/D3 and human breast cancer cell lines; the microsomal fraction of liver; crude membrane fractions of human breast and stomach cancer cell lines; plasma membrane fractions of liver, kidney, platelets, meningioma, hCMEC/D3 cells, and HUVECs; cytosolic fractions of human pancreatic adenocarcinoma cell lines; and the plasma of pancreatic cancer patients [4-7,11-18]. The minimal sample requirement for QTAP experiments is 50 μg protein. The preparation procedures for brain capillaries and plasma membrane fractions are described in section “Preparation of protein samples”.

Step 7 is the preparation of the peptide samples. The protein samples are first denatured with 7 M guanidine hydrochloride or 8 M urea, followed by reduction and S-carbamoylmethylation. When guanidine hydrochloride is used, the alkylated proteins are precipitated with a mixture of methanol, chloroform, and water and dissolved in 6 M urea. When urea is used in the first denaturation step, the precipitation step is omitted. The alkylated proteins are digested with lysyl endopeptidase (LysC), followed by trypsin. Then, a fixed amount of stable-isotope-labeled IS peptides is added, and the sample is acidified. Desalting is performed if needed.

Steps 8 and 9 are the determination of the protein expression levels of the target proteins in the biological samples by LC-MS/MS. The procedures for peak recognition, which is one of the most important steps for accurate quantification, are described in section “Data analysis”.

### Method setup for QTAP

#### Selection of an appropriate peptide from the peptide mixture produced by trypsin digestion of the target protein

The selection of the probe peptide for the target protein is essential to achieve highly reliable and sensitive protein quantification by SRM/MRM analysis. Global proteomic approaches have been applied for peptides obtained by trypsin digestion of biological samples that express high levels of the target protein in which peptides with a high signal intensity are selected for quantification. However, this strategy is not only time-consuming but also requires the preparation of samples that express high levels of the target protein; therefore, the number of proteins for which quantification methods can be established is quite limited. Furthermore, trypsin digestion efficiency, peptide specificity, post-translational modification (PTM), and polymorphisms should be considered for accurate quantification, but the small number of peptides identified by global proteomics does not necessarily allow this.

To solve these problems, we have established a theory to predict the appropriate peptides for SRM/MRM quantification based on previous proteomic data and experience (Table 1). This allowed us to design highly sensitive and highly accurate target peptides *in silico* from sequence information registered in protein databases including UniProtKB [4]. We successfully quantified more than 100 transporter proteins in isolated human brain microvessels [6,19]. We have previously established an LC-MS/MS quantification method for more than 500 proteins including human, monkey, and mouse transporters, enzymes, and receptors, and we have reported quantitative protein expression profiles in brain capillaries, livers, kidneys, platelets, plasma, meningioma, human BBB model cell lines (hCMEC/D3), human umbilical vein endothelial cell lines (HUVECs), human pancreatic adenocarcinoma cell lines, and human breast and stomach cancer cell lines [4-7,11-18]. In contrast to global proteomics, in QTAP, it is important that the target peptides are completely digested by proteases to enable the estimation of the absolute expression levels of the target proteins. Therefore, sequences that may cause incomplete digestion, such as continuous sequences of arginine (R) or lysine (K) (RR, KK, RK, KR) and a proline (P) at the C-terminal side of R or K (RP, KP), and transmembrane regions should be avoided (Table 1). The selection and use of multiple peptides for a target protein is useful for increasing the credibility of the absolute quantification.

Some protein families have a high similarity of amino acid sequences between subtypes, which sometimes makes it difficult to select the specific peptide for each subtype, particularly for short proteins. LysC digestion (digestion at K) is sometimes useful to obtain specific peptides if they cannot be chosen from trypsin-digested peptides (digestion at R and K). Alternatively, a common peptide can be selected for the target and similar proteins, and a specific peptide can be selected for the similar protein. The absolute expression level of the target protein is obtained by subtracting that of the similar protein from the total level, which is determined using the common peptide.

#### Preparation of the peptide solution for the calibration curve and internal standard

For absolute quantification of target proteins, the peptide concentrations of the stock solutions of the non-labeled (standard, St) and stable-isotope-labeled (internal standard, IS) peptides synthesized with > 95% purity must be determined by amino acid analysis (AAA), an accurate quantification method for peptides and proteins. A portion of the stock solution is hydrolyzed for 24 hours at 110°C in 5.7 N HCl to digest the peptide into free amino acids. After the HCl is dried in a vacuum-centrifuge or with N_2_ gas, the resulting amino acids are dissolved in 0.02 N HCl and quantified by an amino acid analyzer (e.g., HPLC-UV system with post-column ninhydrin derivatization) to determine the peptide concentration of the stock solution. The accuracy of the concentration determined by AAA is one of the key factors for accurate absolute quantification of target proteins. Therefore, pipette handling of less than 100 μL volumes should be avoided throughout the experiment, and the experiment should be conducted in quadruplicate at least. After concentration determination, the peptide solution is stored at -80°C. Freeze-thaw cycles should be minimized.

#### Selection of a suitable configuration of mass spectrometer and liquid chromatograph

Table 2 compares the performance of the different types of MS devices available on the market. In tissues and cells, abundant proteins as well as low-abundance proteins play an important functional role. Membrane proteins, such as transporters and receptors, generally have low expression levels. To quantify as many functional proteins as possible, it is necessary to use a MS device with high sensitivity and reliable quantification. Triple quadrupole mass spectrometers (QqQ) enable highly sensitive (attomole) and reliable quantification with a wide dynamic range (six orders of magnitude) by employing the SRM/MRM mode. Recent advances in mass spectrometry technologies have improved the sensitivity and quantification reliability of LTQ, Orbitrap and TOF, so that new generations of these MS instruments can also quantify attomole levels of proteins in SRM/MRM mode. The short dwell time (time spent to acquire the specific SRM/MRM transition) and fast switching of transitions in QqQ permit the simultaneous quantification of hundreds of targets while maintaining acceptable sensitivity. Therefore, we have selected QqQ for QTAP.

**Table 2:** Characteristics of different types of mass spectrometers

**Instrument**	**Characteristics**				**Performance for proteomics**	
	**Ion source**	**Mass resolution**	**Sensitivity**	**Dynamic range**	**Identification**	**Quantification**
QIT, LTQ	ESI & MALDI	Low to medium	Medium	Narrow to medium	Medium	Medium
QqQ	ESI	Low	High	Very wide	Low	Very high
TOF-TOF	MALDI	Medium	Medium	Narrow	High	Low
Q-TOF	ESI & MALDI	Medium	Medium	Medium	High	High
LTQ-Orbitrap	ESI & MALDI	Medium to high	Medium	Narrow to medium	High	Medium
FTICR	ESI & MALDI	Very high	Medium	Very narrow	High	Very low

Recent MS instrument development has improved the quantification performance of high-resolution MS devices. The latest Q-TOF (AB Sciex TripleTOF 5600) can achieve highly sensitive target quantification with a dynamic range of four to five orders of magnitude while maintaining high resolution, which is referred to as high-resolution SRM/MRM (HR-SRM/MRM) analysis. Therefore, this MS device significantly reduces the noise level observed in the traditional SRM/MRM mode and can quantify target peptides with small peaks that are masked by background noise in QqQ. Furthermore, the improved scan speed of the MS device permits multiple transition analysis for the quantification of several target peptides. Therefore, this MS device would be useful for the quantification of target proteins in highly complex protein samples, such as whole tissue lysates, that have high levels of background noise.

LC selection is also important for QTAP. Conventional HPLC and nanoLC separation prior to MS analysis are the most widely used separation methods. The advantages of conventional HPLC are that it is more robust and easier to use, even for beginners, and that larger amounts of sample can be analyzed compared to nanoLC. The disadvantage of conventional HPLC is that it is less sensitive than nanoLC separation, thereby requiring a small ID column such as a 1.0 mm ID C18 column with a flow rate of 50 μL/min. By contrast, because nanoLC separation is sensitive, the analysis can be performed with low amounts of sample. For example 1 μg trypsin-digested peptides can be analyzed in sample-limited situations; however, large amounts of sample cannot be injected due to the small column ID and flow path. However, the nanoLC system is less robust and difficult to use, which requires training and patience, leading to low reproducibility. Recently, the introduction of a nanoLC system coupled with a chip column, e.g., the Eksigent NanoLC-Ultra and Ekspert nanoLC400, has led to high robustness and ease-of-use for nanoLC-MS/MS. Chip columns can be exchanged in seconds and provide reproducible results day-to-day, column-to-column and lab-to-lab.

The extension of the upper pressure limit of HPLC instrumentation to 1300 bar (ultra-high pressure liquid chromatography, UHPLC) and the introduction of columns that are packed with porous sub-2-μm and superficially porous (fused-core or core-shell) particles have opened new frontiers in the resolution of target peaks and analysis speed. Small-diameter ESI electrodes, such as 25 and 50 μm ID, have been developed and can minimize post-column dispersion, leading to sharper peaks. Therefore, microflow UHPLC-MS/MS with a superficially porous column and a small-diameter ESI electrode achieves more sensitive and higher throughput quantification than conventional HPLC-MS/MS. Furthermore, the microflow UHPLC system is as robust as conventional HPLC. However, the narrow peaks that are produced by microflow UHPLC (peak width less than 10 seconds) require a fast duty cycle in the MS device, which is only available in the latest generations of MS devices. Certain MS analyzers with fast acquisition speed (e.g., QqQ or TOF) are more compatible with microflow UHPLC than others (e.g., ion trap, Orbitrap, or FT-ICR).

#### Setup of LC-MS/MS quantification system for SRM/MRM analysis

Here, we describe the principle of SRM/MRM analysis and how to optimize the analytical conditions for peptide quantification in the SRM/MRM mode of HPLC-QqQ, which is one of the most routinely used configurations of LC and MS for quantification.

**Figure 3:**
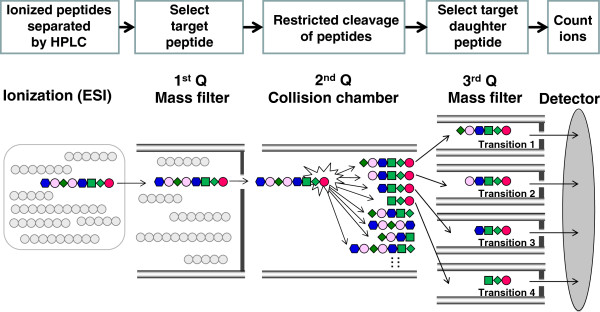
**Principle of peptide selection by selected/multiple reaction monitoring (SRM/MRM) mode of triple quadrupole mass spectrometry (QqQ MS).** The selection of the targeted peptide by two mass filters (Q1 and Q3) results in the reduction of noise from the complex peptide sample. Each target peptide is monitored by four different SRM/MRM transitions, which consist of a parent ion (Q1) and four different daughter ions (Q3) for accurate and reliable quantification.

The target peptide is quantified by SRM/MRM mode using QqQ to achieve highly selective and sensitive quantification (Figure 3). QqQ employs three chambers; the 1st Q (Q1) and 3rd Q (Q3) are mass filters that pass the peptide ion with the target mass. In the 2nd Q (Q2), the peptide ion is fragmented by collision with N_2_ gas. The use of two mass filters provides high selectivity and a high S/N ratio. The combination of Q1 and Q3 mass filters is called an SRM/MRM transition, which can be changed every few msec, and hundreds of SRM/MRM transitions can be simultaneously monitored in a single analysis.

**Figure 4:**
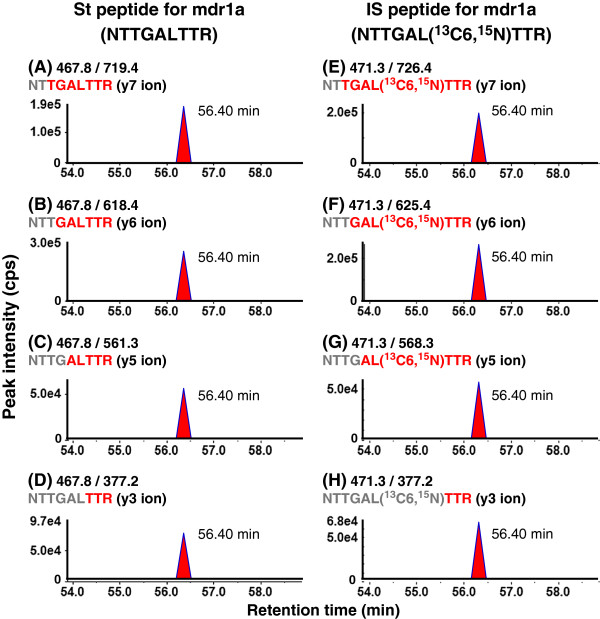
**Typical SRM/MRM chromatograms of a standard and internal standard peptide to make a calibration curve.** The St peptide mixture (500 fmol each) and IS peptide mixture (500 fmol each) were subjected to LC-MS/MS and analyzed in SRM/MRM mode under optimized analytical conditions. **A-D**: SRM/MRM chromatograms of four transitions for the St peptide of mdr1a (NTTGALTTR). **E**-**H**: SRM/MRM chromatograms of four transitions for the IS peptide of mdr1a (NTTGAL(^13^C6,^15^ N)TTR).

**Figure 5:**
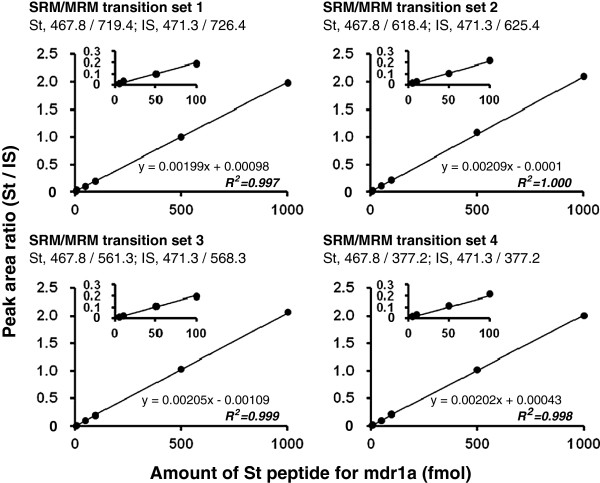
**Typical calibration curve for absolute quantification.** Dilution series of the St peptides (5, 10, 50, 100, 500 and 1000 fmol) and 500 fmol IS peptides were subjected to LC-MS/MS and analyzed in SRM/MRM mode under optimized analytical conditions. The calibration curves were prepared using every transition set by plotting the peak area ratios of the St and IS peptides (y-values) against the St peptide amounts (x-values). This figure represents the calibration curve for mdr1a. *R*^*2*^, correlation coefficient.

In our approach, each target peptide is quantified by measuring four different SRM/MRM transitions, which consist of a parent ion (Q1) and four different daughter ions (Q3). This allows us to increase the selectivity for the target peptide by monitoring the chromatographic coelution of eight transitions of the target and internal standard peptides, thereby ensuring the reliable identification of signal peaks (Figure 4). Furthermore, this SRM/MRM analysis provides four quantitative values for a target peptide from four transition sets by using the four corresponding calibration curves (Figure 5), increasing the accuracy of the quantification. By comparing the four quantitative values, the overlap of the noise peaks with the target peptide peak can be determined, and when necessary, SRM/MRM transitions can be changed to appropriate transitions that are not affected by noise peaks. A total of 8 SRM/MRM transitions (four transitions for the target peptide and four corresponding transitions for the internal standard peptide) are required for the quantification of one protein. Therefore, 37 different proteins can be simultaneously quantified in a single analysis using the currently available maximum of 300 SRM/MRM transitions (multiplexed SRM/MRM analysis).

To achieve a highly sensitive SRM/MRM analysis for individual peptides, it is essential to select four highly sensitive transitions and to optimize the declustering potentials (DP) and collision energies (CE). These conditions are determined from MS/MS spectra that are obtained by the direct infusion with a syringe pump of a 0.1-1 μM peptide solution at a flow rate of 5 μL/min into the MS device. Typically, doubly charged precursor ions (singly or triply charged for some peptides) are selected (Q1). A total of four transitions per peptide (Q3-1, -2, -3 and -4) that correspond to high intensity daughter ions are selected. The DP and CE are optimized to maximize signal strength. For the internal standard peptides that are labeled with ^13^C and/or ^15^N, the 4 transitions corresponding to those of the standard peptides are selected with the same DP and CE as the standard peptides. A dilution series of standard peptide (blank, 1, 5, 10, 50, 100, 500 and 1000 fmol) with a fixed amount of internal standard peptide (500 fmol for AB Sciex API5000; 100 fmol for AB Sciex QTRAP5500) is injected onto the C18 column of the LC coupled with the QqQ to confirm the appropriate separation of the peptide by the column and the sensitivity and accuracy under the optimized analytical conditions of SRM/MRM analysis (Figures 4 and 5). Table 3 shows an example of the optimized conditions for SRM/MRM analysis using an API5000 and a QTRAP5500. Peptides can be simultaneously quantified under optimized conditions in SRM/MRM mode.

**Table 3:** An example of optimized analytical conditions for multiplexed SRM/MRM analysis in API5000 and QTRAP5500

**Molecule name**	**AA Seq.**	**St/IS**	**Transition number**	**Q1 m/z**	**Q3 m/z**	**Dwell time (msec)**	**DP**	**CE**
Mdr1a	NTTGALTTR	St	1	467.8	719.4	10	51	30
			2	467.8	618.4	10	51	30
			3	467.8	561.3	10	51	30
			4	467.8	377.2	10	51	30
	NTTGAL*TTR	IS	1	471.3	726.4	10	51	30
			2	471.3	625.4	10	51	30
			3	471.3	568.3	10	51	30
			4	471.3	377.2	10	51	30
Tfr1	SSVGTGLLLK	St	1	487.8	800.5	10	56	25
			2	487.8	701.5	10	56	23
			3	487.8	644.4	10	56	20
			4	487.8	543.4	10	56	21
	SSVGTGLLL*K	IS	1	491.3	807.5	10	56	25
			2	491.3	708.5	10	56	23
			3	491.3	651.4	10	56	20
			4	491.3	550.4	10	56	21
Na^+^/K^+^-ATPase	AAVPDAVGK	St	1	414.2	685.4	10	50	17
			2	414.2	586.3	10	50	17
			3	414.2	489.3	10	50	27
			4	414.2	374.2	10	50	27
	AAVPDAV*GK	IS	1	417.2	691.4	10	50	17
			2	417.2	592.3	10	50	17
			3	417.2	495.3	10	50	27
			4	417.2	380.2	10	50	27

Bold letters with asterisks indicate amino acid residues that are labeled with a stable isotope (^13^C and ^15^ N).

Abbreviations: *AA* amino acid; *St* standard; *IS* internal standard; *DP* declustering potential; *CE* collision energy.

The other MS parameters, including those that are related to the ESI source, are common between peptides, and the optimized values for HPLC-API5000 or QTRAP5500 with a flow rate of 50 μL/min are listed in Table 4. Polarity switching between positive and negative ESI is useful to prevent decreased MS sensitivity caused by the accumulation of ions inside of the analyzer. Thus, a positive mode is followed by a negative mode for a short period after the elution of the target peptides (Table 4). The HPLC conditions are also listed in Table 4. The gradient profile for HPLC is set at approximately 1%B/min or less than 1%B/min to avoid significant ion suppression. The switching valve enables the removal of undesired substances, including salts. Therefore, the valve is essential to keep the MS analyzer clean and to avoid decreased sensitivity.

**Table 4:** Analytical condition of HPLC-API5000 or QTRAP5500 systems for peptide quantification

		
**Mass spectrometer (API5000, QTRAP5500)**	Turbo V ion source, SRM/MRM mode, total duration: 120 min.	
	Period 1 (100 min):	Positive ionization, CAD 12, CUR 40, GS1 20, GS2 40, IS 5500, TEM 500, ihe ON, EP 10, CXP 12.
	Period 2 (20 min):	Negative ionization, CAD 12, CUR 40, GS1 20, GS2 40, IS -4500, TEM 500, ihe ON, EP -10, CXP -12.
**HPLC (Agilent Technologies 1200 Series)**	Column:	Waters XBridge BEH130 C18 (1.0 × 100 mm, 3.5 μm) column (Waters, Cat. No. 186003561) connected to a guard column (2.1 × 10 mm, 3.5 μm, Waters, Cat. No. 186003059) and a Sentry 2.1 × 10 mm guard holder (Waters, Cat. No. WAT097958).
	Mobile phases:	A and B consist of 0.1% formic acid in water and 0.1% formic acid in acetonitrile, respectively.
	Pump:	Peptides are separated and eluted from the column at 40°C (column oven) using a linear gradient with a 120 min run time at a flow rate of 50 μL/min. The sequence is as follows:
		0 min: 99% A, 1% B
		5 min: 99% A, 1% B
		60 min: 40% A, 60% B
		61 min: 0% A, 100% B
		63 min: 0% A, 100% B
		65 min: 99% A, 1% B
		120 min: 99% A, 1% B
	Autosampler:	Injection volume, 40 μL. Sample loop, 100 μL. Temperature, 10°C. Needle wash with 0.1% formic acid in water for 5 sec. Well bottom sensing to inject the exact volume.
	Valve (after HPLC column and before mass spec):	0-20 min: directed to waste.
		20-120 min: directed to mass spec ion source.

### Preparation of protein samples

#### Preparation of brain capillary-rich fraction (whole tissue lysate)

**Figure 6:**
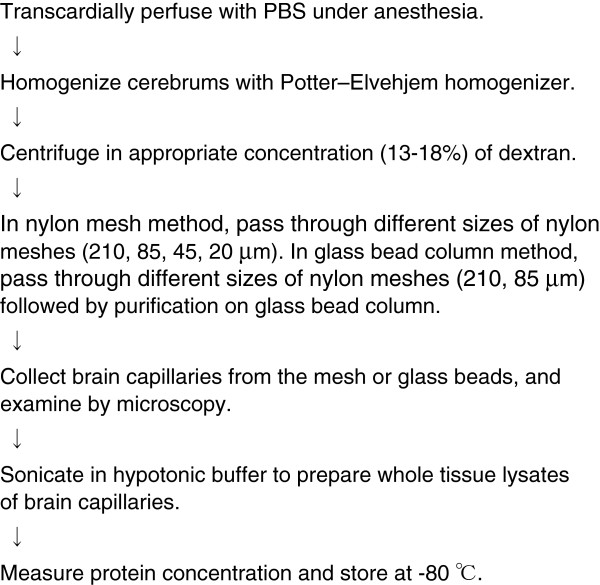
Brain capillary isolation procedure.

Figure 6 shows a preparation procedure for the brain capillary-rich fraction. The details for this procedure have been described previously [6,20,21]. Here, we describe the precautions that should be followed for this experiment:

1. Homogenization: A Potter–Elvehjem homogenizer with a medium clearance should be used to avoid disrupting endothelial cells. Manual up-and-down strokes without rotation are essential to prevent the aggregation of brain capillaries. The number of strokes should be optimized in advance to achieve a high purity and recovery of brain capillary.

2. Centrifugation with dextran: the appropriate concentration of dextran to isolate brain capillaries with high purity and recovery varies depending on the animal, the state of the brain (fresh, frozen, different degrees of freshness) or dextran lot [5,6]. Therefore, the concentration should be optimized in advance.

3. Different sizes of nylon meshes (210, 85, 45, 20 μm) are used to fractionate brain vessels. The vessels passing through a 85-μm mesh are brain capillaries. Nylon mesh or glass beads should be washed well after trapping brain capillaries to ensure blood cell removal.

4. The nylon mesh method is more appropriate than the glass bead column method for isolating capillaries from frozen brain.

5. After collecting capillaries from mesh or beads, the frequency of pipetting should be minimized to avoid the adsorption of capillaries to the pipette tip.

6. Freeze-thaw cycles of capillaries should be minimized.

7. The recovery of brain capillary is approximately 50–100 μg protein from whole capillary lysate/g brain. Because the minimal requirement for QTAP is 50 μg protein/sample, it is recommended to prepare brain capillaries from at least 3 g brain (10 mouse or 3 rat cerebrums) because a significant loss of capillaries could occur throughout the experiment if the starting amount of brain is small.

#### Preparation of plasma membrane fraction

**Figure 7:**
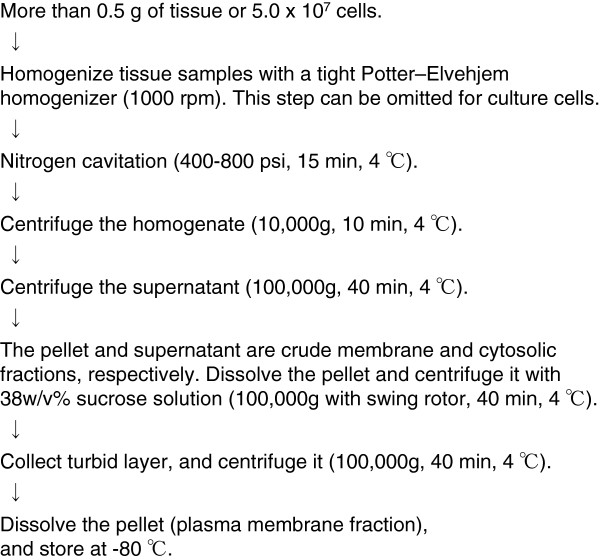
Preparation procedure of plasma membrane fraction.

One of the advantages of quantification at the protein level is that the expression levels of target proteins in certain subcellular compartments can be determined by subcellular fractionation. This is crucially different from quantification at the mRNA level. Recently, we demonstrated that the protein expression levels of transporters in the plasma membrane fraction do not correlate with their mRNA expression levels in 17 human liver biopsies [16], thereby highlighting the advantage of quantification at the protein level, particularly in the plasma membrane fraction, to understand the transport activities of membrane transporters. For QTAP analysis using the plasma membrane fraction, it is important to prepare the sample with highly reproducible purity and recovery. Figure 7 shows a plasma membrane fraction preparation procedure. The details for the procedure have been described previously [4,7,16]. Here, we describe the precautions that should be followed to achieve high reproducibility:

1. At least 0.5 g tissue or 5.0 × 10^7^ cells are needed to stably obtain more than 100 μg protein from the plasma membrane fraction. To prepare the plasma membrane fraction of isolated brain capillaries, at least 50 g brain tissue is needed.

2. After nitrogen cavitation, cell disruption should be confirmed by microscopy. If disruption is insufficient, the samples should be homogenized or subjected to nitrogen cavitation again. The pressure that is used for nitrogen cavitation can also be increased.

3. The sample suspension should be gently stacked on top of a pre-established 38% sucrose solution to avoid disturbing the interface between the sample suspension and the 38% sucrose solution. After ultra-centrifugation, the turbid layer should be carefully recovered with a syringe.

4. Protease inhibitors should be used throughout the experiment. For phosphoproteomics, phosphatase inhibitors should be used in addition to protease inhibitors.

5. Freeze-thaw cycles of the plasma membrane fraction should be minimized.

### Absolute quantification by LC-MS/MS

#### Materials

The materials that are used for QTAP are listed in Table 5.

**Table 5:** Materials for QTAP

**Name**	**Company**	**Catalog#**	**Amount**	**Note**
Guanidine HCl	Wako	070-01825	500 g	For denaturing buffer
2NA (EDTA·2Na)	DOJINDO	345-01865	500 g	For denaturing buffer
Trizma® (Tris) base, primary standard and buffer	SIGMA	T1503-1KG	1 kg	For several buffers
Ammonium hydrogencarbonate	Wako	017-02875	500 g	For LysC and ProteaseMax solution
(±)-Dithiothreitol (DTT)	Wako	049-08972	25 g	
Iodoacetamide (IAA)	Wako	093-02152	25 g	
Chloroform	Wako	038-02606	500 mL	
Methanol	Wako	137-01823	3 L	
Urea	Wako	211-01213	1 kg	
ProteaseMax surfactant, trypsin enhancer	Promega	V2072	5 × 1 mg	
Lysyl endopeptidase (LysC)	Wako	129-02541	10 AU	
Sequencing grade modified trypsin, frozen (TPCK-trypsin)	Promega	V5113	100 μg	
SUMILON proteosave SS, 1.5 mL tube	SUMITOMO BAKELITE	MS-4215 M	100/pk	Low protein binding tube
SUMILON proteosave SS, 0.5 mL tube	SUMITOMO BAKELITE	MS-4205 M	100/pk	Low protein binding tube
Ultra plus tip 1–10 μL	bms	UP-0110	96/pk	Low protein binding tip
Ultra plus tip 1–200 μL	bms	UP-2010	96/pk	Low protein binding tip
Eyela cute mixer CM-1000	TOKYO RIKAKIKAI	188140		Tube mixer
Bransonic ultrasonic cleaner 2510 J-DTH	Branson	2510 J-DTH		Sonicator
High speed refrigerated micro centrifuge MX-160	TOMY			Centrifuge
TMA-30	TOMY			Angle rotor
TMS-21	TOMY			Swing rotor
Block incubator BI-525	ASTEC			LysC digestion
Incubator MIR-262	SANYO	MIR-262		Trypsin digestion
Formic acid (98.0%)	Wako	066-00466	500 mL	Acidification
Acetonitrile (LC/MS grade)	Wako	018-19853	3 L	Mobile phase
XBridge BEH130 C18 (1.0 × 100 mm, 3.5 μm) column	Waters	186003561		HPLC column
XBridge C18 Guard Cartridge (2.1 × 10 mm, 3.5 μm)	Waters	186003059	2/pk	Guard column
Sentry 2.1 mm guard holder	Waters	WAT097958		Guard column holder
Agilent technologies 1200 series (HPLC system)	Agilent			HPLC
API5000	AB Sciex			QqQ mass spectrometer
QTRAP5500	AB Sciex			QqQ mass spectrometer
96 well plates, 0.5 mL, polypropylene	Agilent	5042-1386	10/pk	Sample plate for auto sampler (HPLC)
Pre-slit well cap for 96 well PP plate non sterile, silicone	Thermo SCIENTIFIC	276011	10/pk	Sample plate cap for auto sampler (HPLC)
DC protein assay reagent A	BIO-RAD	500-0113	250 mL	Protein quantification (Lowry method)
DC protein assay reagent B	BIO-RAD	500-0114	1 L	Protein quantification (Lowry method)

#### Reagent setup

The reagents that are used for QTAP are listed in Table 6 with compositions, storage temperature, and other information.

**Table 6:** Reagents used for QTAP

**Reagent**	**Composition**	**Storage temp.**	**Note (how to prepare, store and use)**
Denaturing buffer	500 mM Tris–HCl (pH 8.5), 7 M guanidine HCl, 10 mM EDTA	R.T.	Stir solution at 50°C to dissolve guanidine HCl.
DTT solution	50 μg/μL dithiothreitol (DTT)	-	Should be freshly prepared and used within 1 h.
IAA solution	50 μg/μL iodoacetamide (IAA) in denaturing buffer	-	Should be freshly prepared in denaturing buffer, protected from light and used within 1 h.
Tris–HCl buffer	0.1 M Tris–HCl (pH 8.5)	R.T.	-
Urea solution	6 M urea, 0.1 M Tris–HCl (pH 8.5)	-	Should be freshly prepared.
LysC solution	0.5 μg/μL lysyl endopeptidase (LysC; Wako 129–02541)	-80°C	Should be divided into single-use aliquots (e.g., 5 μL/tube) and stored at -80°C until use.
ProteaseMax solution	1% w/v ProteaseMax (Promega V2072)	-20°C	Should be divided into single-use aliquots (e.g., 10 μL/tube) and stored at -20°C until use.
Trypsin solution	0.5 μg/μL TPCK-trypsin (Promega V5113)	-80°C	Should be divided into single-use aliquots (e.g., 5 μL/tube) and stored at -80°C until use.
Peptide mixture	100 nM St or IS peptide mixture	-80°C	Mix stock solutions of different peptides so that each peptide concentration is 100 nM. Should be divided into single-use aliquots (e.g., 50 μL/tube) and stored at -80°C until use.

Abbreviations: *R.T*. room temperature; *St* standard; *IS* internal standard.

#### Preparation of peptide samples (proteins to peptides)

Peptide samples are prepared according to the procedure described in Table 7. First, proteins (50–100 μg) are solubilized, denatured with denaturing buffer, reduced by DTT, and S-carbamoylmethylated by IAA. Second, the alkylated proteins are precipitated with a mixture of methanol, chloroform and water. Third, the protein precipitates are dissolved in 6 M urea in 100 mM Tris–HCl (pH 8.5), diluted 5-fold with 100 mM Tris–HCl (pH 8.5), and digested with LysC followed by digestion with TPCK-treated trypsin.

**Table 7:** Sample preparation procedure for LC-MS/MS analysis

	**Procedure**	**Notes**
**I. Reduction and alkylation of proteins**		
1.	Add denaturing buffer to 50 μg protein of protein sample on ice (total volume should be 220 μL).	· Denature protein.
		· Can deal with samples at r.t. after denaturing protein samples.
		·Should use low-protein-adsorption 1.5-mL tubes, e.g., SUMITOMO BAKELITE, SUMILON Proteosave SS 1.5 mL tubes, MS-4215 M.
		· Do not pipet to prevent adsorption of proteins in pipette tips.
2.	Add same amount of DTT as protein amount (Add 1 μL of 50 μg/μL DTT solution).	· Do not pipet.
3.	Stir the sample using a tube mixer (e.g., cute mixer CM-1000, EYELA) for 60 min at r.t.	· Reduction of S-S bond.
4.	Add 2.5-fold IAA of protein amount (Add 2.5 μL of 50 μg/μL IAA solution).	· Do not pipet.
5.	Stir the sample using a tube mixer for 60 min at r.t. in the dark.	· Protection of –SH residue (alkylation)
		· IAA can be degraded by light, so the sample tubes should be protected from light.
**II. Methanol-chloroform precipitation (on ice)**		
6.	Add 600 μL cold methanol to sample solution. Invert the tube.	· Do not pipet.
7.	Add 150 μL cold chloroform to sample solution. Invert the tube.	· Do not pipet.
8.	Immediately after adding 450 μL cold water to sample solution and inverting the tube, centrifuge the sample using swing rotor at 15,000 rpm for 5 min at 4°C.	· Do not pipet.
9.	Immediately after centrifugation, remove the upper layer (until a floating pellet).	· The floating pellet is protein.
		· Do not take the protein pellet.
10.	Add 450 μL cold methanol to sample solution. Invert the tube gently to wash the protein pellet.	· Do not pipet.
11.	Centrifuge sample using swing rotor at 15,000 rpm for 5 min at 4°C.	
12.	Immediately after centrifugation, remove the supernatant.	· Do not take the protein pellet.
13.	Again centrifuge sample using swing rotor at 15,000 rpm for 1 min at 4°C and remove the supernatant completely.	· Do not take the protein pellet.
**III. Double digestion with LysC and trypsin**		
14.	Add 9 μL 6 M urea solution, and stir the sample using tube mixer for approximately 10 min at r.t.	· Do not pipet.
15.	Add 36 μL 0.1 M Tris–HCl buffer (pH 8.5).	· Final concentration of urea is 1.2 M.
		· Do not pipet.
16.	Resuspend protein pellet by intermittent sonication with Branson 2510 sonicator.	· Sonication for 30 seconds followed by a pause for 30 seconds on ice. Repeat this step until the pellet is resuspended.
17.	Add 1/100-fold LysC of the protein amount (add 1 μL 0.5 μg/μL LysC solution).	· Do not pipet.
		· Gently tap using finger to stir sample solution.
18.	Add 1% ProteaseMax solution (2.5 μL) so that the final concentration is 0.05%.	· Do not pipet.
		· Gently tap using finger to stir sample solution.
19.	Incubate sample at 25°C for 3 h.	
20.	Add 1/100-fold TPCK-trypsin of the protein amount (Add 1 μL of 0.5 μg/μL TPCK-trypsin solution).	· Do not pipet.
		· Gently tap using finger to stir sample solution.
21.	Incubate sample at 37°C for 16 h.	· Total volume is 49.5 μL.
**IV. LC-MS/MS analysis**		
22.	Add 7.5 μL of IS peptide mixture.	· The concentration of the IS peptide mixture should be adjusted so that the injected amount of each peptide is 500 fmol for HPLC-API5000 or 100 fmol for HPLC-QTRAP5500.
		· Pipet well in sample solution when adding IS peptide solution, then mix with vortex mixer.
23.	Add 3 μL 50% formic acid in water.	· Acidification.
		· Mix with vortex mixer.
		· Total volume is 60.0 μL.
24.	Centrifuge sample at 15,000 rpm for 5 min at 4°C with an angular rotor.	
25.	Apply 58 μL supernatant to 96-well plates or vials in autosampler. Keep the autosampler at less than 10°C.	
26.	Inject 40 μL on LC-MS/MS.	· 40 μL includes 33.3 μg peptide sample (50 μg protein × 40 μL/60 μL) and 100 fmol (QTRAP5500) or 500 fmol (API5000) of IS peptides.

**Figure 8:**
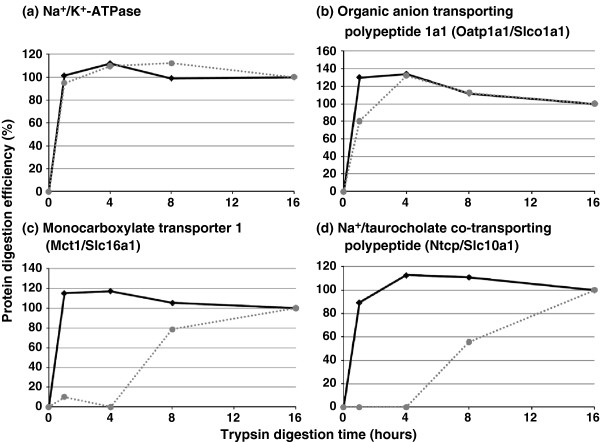
**Efficiency of enzymatic digestion of plasma membrane proteins: Na**^**+**^**/K**^**+**^**-ATPase (a), Organic anion transporting polypeptide 1a1 (b), Monocarboxylate transporter 1 (c), and Na**^**+**^**/taurocholate co-transporting polypeptide (d) in mouse liver.** Plasma membrane proteins of mouse liver were digested with only trypsin for the indicated time at 37°C (dotted line) and with lysyl endopeptidase C for 3 h at 25°C, followed by trypsin for the indicated time at 37°C (solid line). The digestion efficiency (%) was calculated by the following equation: [the absolute amounts of digested peptides at the indicated time] × 100/[the absolute amounts of digested peptides by the 16h digestion].

The efficiency of enzyme digestion is one of the key points for the absolute quantification of target proteins. We have confirmed the efficient digestion of glut1 in mouse brain microvessels and human MDR1 in MDR1-overexpressing cells by comparing the absolute amounts of digested peptides with that determined by quantitative binding assay and immunoblotting [4]. Furthermore, we have also confirmed that no bands greater than 20 kDa were observed by SDS-PAGE after trypsin digestion [4]. These results suggest that enzyme digestion proceeds efficiently; however, the results do not necessarily indicate complete digestion for all molecules other than glut1 and MDR1. As shown in Figure 8, the digestion speed differs between molecules, suggesting that the time dependency of trypsin digestion should be examined before the absolute quantification of target proteins to determine the digestion efficiency. In case of inefficient digestion, it is necessary to change target peptides and/or optimize the digestion reaction condition. Figure 8 also shows a dramatic improvement of the digestion rate of monocarboxylate transporter 1 (Mct1/Slc16a1) and Na^+^/taurocholate co-transporting polypeptide (Ntcp/Slc10a1) by a combination of LysC, trypsin, and a trypsin enhancer (ProteaseMax) compared to single digestion with trypsin. Therefore, the use of LysC and ProteaseMax in addition to trypsin is useful to facilitate enzyme digestion.

#### LC-MS/MS analysis

After trypsin digestion, a fixed amount of IS peptides is added to the digested peptide samples and a dilution series of St peptides (blank 1, 5, 10, 50, 100, 500 and 1000 fmol as injected amounts) prepared from a 100 nM St peptide mixture; then, the digested peptide samples and dilution series are acidified and centrifuged (Table 7). The supernatants are analyzed under the optimized analytical condition (Tables 3 and 4). Each target peptide is measured by 4 different SRM/MRM transitions (Figure 4), and up to 37 proteins can be simultaneously quantified. If a scheduled SRM/MRM mode is applied, it is possible to simultaneously quantify more than 37 proteins. The time required for a single analysis is 2 hours using a traditional HPLC-MS/MS system because a slow gradient is necessary to avoid significant ion suppression, and thus an entire analysis, including the dilution series, target protein samples, and quality controls, would be finished within a few days.

The sample solution obtained by the trypsin digestion of biological samples includes not only peptides but also various substances, including salts from “dirty” samples, that can contaminate the MS device and decrease sensitivity. Therefore, the clean-up of samples prior to introduction to the MS device is crucial to maintain its performance. Valve switching after the C18 column and before the MS device is useful to automatically remove the salts and hydrophilic substances (Table 4). Furthermore, the clean-up of samples with desalting tips prior to LC-MS/MS injection is also useful to avoid sample clogging in the column and flow path, particularly in nanoLC, while keeping the MS device cleaner and concentrating peptide samples before injection. GL-Tip SDB (GL Sciences Inc., 200 μL, 7820–11200) is superior to conventional C18 desalting tips for the retention of more hydrophilic peptides; in addition, this device eliminates the loss in the desalting step of low hydrophobic peptides that are retained on the C18 HPLC analytical column and not retained in C18 desalting tip. The GL-Tip GC (GL Sciences Inc., 200 μL, 7820–11201) can cover a wider hydrophilic range of peptides than the GL-Tip SDB. Therefore, the combined use of the GL-Tip SDB and the GL-Tip GC is beneficial for covering a wide range of hydrophilic and hydrophobic peptides.

#### Data analysis

Data analysis includes peak recognition, calibration curve preparation, and protein expression level and limit of quantification calculations. Here, we describe the precautions that should be followed for data analysis.

### Peak recognition

**Figure 9:**
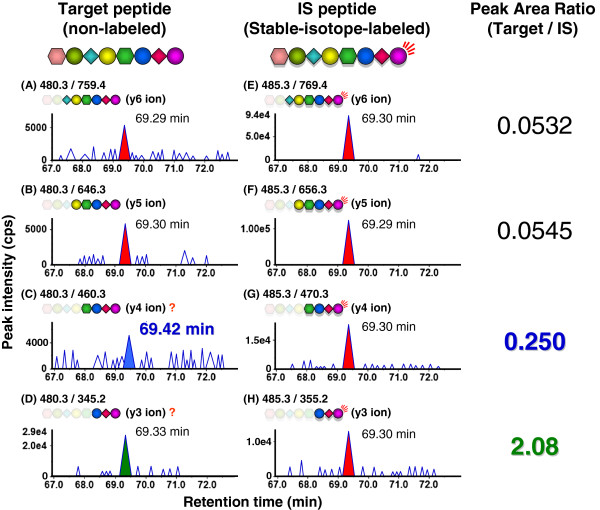
**An example of ambiguous SRM/MRM chromatograms of a biological sample.** Tryptic digests of a biological protein sample spiked with IS peptides were subjected to LC-MS/MS and analyzed in SRM/MRM mode. **A-D**: SRM/MRM chromatograms of four SRM/MRM transitions for a target (non-labeled) peptide. **E-H**: SRM/MRM chromatograms of four SRM/MRM transitions for the corresponding IS peptide.

A positive peak for the target peptide is defined as one that is detected at the same retention time (± 0.10 min) as the IS peptide under the HPLC conditions that are listed in Table 4. Because a target peptide and its corresponding IS peptide are monitored by 4 transitions, the peak recognition is confirmed by the chromatographic coelution of the 8 transitions (Figure 4). Figure 9 illustrates an example of an ambiguous chromatogram. The retention time of the peak in Figure 9C is more than 0.10 min different from that of the IS peptide in Figure 9G. Therefore, the peak is not derived from the target peptide and should not be recognized. Furthermore, the peak area ratios of the target and IS peptides are theoretically identical between the four transition sets (Figure 4). As shown in transition sets 3 and 4 (Figure 9C, D, G and H), if certain transition sets display larger peak area ratios than other transition sets, it is possible that the recognized peaks include noise peaks. We have previously established that the coefficients of variation for protein expression levels of various proteins were within 20% when determined from three transition sets [4]. Therefore, in principle, we consider that coefficients of variation of over 20% among the four transition sets are likely to indicate the presence of noise peaks, and the corresponding transitions should not be used for accurate quantification. To solve these problems, it is beneficial to use a high-resolution MS device, such as a TripleTOF5600 (Table 2), or to change the target peptide.

### Preparation of the calibration curve

Calibration curves are prepared with every transition by plotting the peak area ratios of the St and IS peptides (y-values) against the St peptide amounts (x-values) (Figure 5). The correlation coefficient (R^2^) of the regression line should be greater than 0.99 for accurate quantification. The slope of the line should be [1/IS peptide amount]. Otherwise, the peptide concentration determined by AAA may be incorrect, and/or the peptides may have adsorbed to the tubes and pipette tips during the preparation of the dilution series. The precision of the St peptide concentration is more critical than that of the IS peptide because an incorrect concentration of the St peptide will result in the incorrect calculation of the protein expression levels of the target proteins.

### Calculation of protein expression level

Kamiie et al. [4] have validated that protein expression levels yield coefficients of variation of less than 20.0% when determined from three peaks with area counts greater than 5000. Therefore, signal peaks with an area count greater than 5000 that are detected at the same retention time (± 0.10 min) as an IS peptide are defined as positive. When positive peaks are observed in three or four sets of transitions, the proteins are considered to be expressed in the target protein samples. The absolute amount (fmol) of each target peptide is determined as the average of three or four quantitative values that are calculated from the target-to-IS peptide peak area ratios in the target samples and the calibration curve. The protein expression level (fmol/μg protein) of the target protein is obtained by dividing the determined absolute amount (fmol) of the target peptide by the total protein amount (μg protein) of the analyzed samples. Biological samples, such as tissues and cells, suffer from significant ion suppression compared to authentic samples, not including the matrix, resulting in a 2- to 10-fold decrease in the peak area of peptides in the biological samples compared with the authentic samples. The degree of ion suppression also differs between samples. Therefore, sensitivity correction using IS peptides is important for the accurate quantification of target proteins.

### Calculation of the limit of quantification (LQ)

The LQ of non-detected molecules in protein samples is defined as the protein concentration (fmol/μg protein) that yields a peak area count of 5000 in the chro-matogram of the target protein samples. When the calibration curve is obtained using Eq. 1, the amount (fmol) of target protein that is equivalent to a peak area count of 5000 (A_Target eq 5000_) is calculated using Eq. 2 from the peak area (counts) of the IS peptide in the target protein samples (PA_IS__in sample_) and the values of Slope and Intercept in Eq. 1. Then, the LQ is obtained with Eq. 3 by dividing A_Target eq 5000_ by the total protein amount (μg protein) of the target protein samples analyzed (A_Sample_).

(1) $$\begin{array}{c} {\mathrm{PA}}_{\mathrm{St}\;\mathrm{in}\;\mathrm{Authentic}}/{\mathrm{PA}}_{\mathrm{IS}\;\mathrm{in}\;\mathrm{Authentic}}=\mathrm{Slope}\times A_{\mathrm{St}\;\mathrm{in}\;\mathrm{Authentic}}\\ \;+\mathrm{Intercept} \end{array}$$

(2) $$A_{\mathrm{Target}\;\mathrm{eq}\;5000}=\left(5000\;\mathrm{counts}/{\mathrm{PA}}_{\mathrm{IS}}{}_{\mathrm{in}\;\mathrm{sample}}–\mathrm{Intercept}\right)/\mathrm{Slope}$$

(3) $$\mathrm{LQ}=A_{\mathrm{Target}\;\mathrm{eq}\;5000}/A_{\mathrm{Sample}}$$

where PA_St in Authentic_ and PA_IS in Authentic_ are the peak areas (counts) of the St peptide and IS peptide in the authentic samples, respectively, and A_St in Authentic_ is the amount (fmol) of the St peptide in the authentic samples. *In silico* peptide selection criteria enable the selection of highly sensitive peptides for any proteins. As a result, the LQs have been reported to be less than 1 fmol/μg protein for 99% of the transporters and receptors in the analysis of human brain microvessels [6].

#### Software for data analysis

The simultaneous quantification of many proteins results in a significant amount of SRM/MRM data. For example, the simultaneous quantification of 37 proteins uses 296 SRM/MRM transitions per analysis (37 × 8 (4 transitions for the target peptide and 4 transitions for the IS peptide)), and the SRM/MRM analysis of 20 samples including eight standards (blank, 1, 5, 10, 50, 100, 500 and 1000 fmol) and 12 biological samples results in 5920 SRM/MRM chromatograms (296 × 20). Therefore, the rapid recognition of target peaks and the rapid calculation of protein expression levels are essential. Conventional software for data processing includes an automatic peak recognition function that is based on the retention time of a signal peak in certain sample analyses. However, the different retention times of signal peaks for peptides between samples make it difficult to properly recognize the target peaks. As a result, the target peaks must be manually recognized in most chromatograms, which is labor-intensive and requires approximately one week for data analysis. To solve this problem, we have developed automatic analysis software that is specialized for simultaneous absolute quantification when stable-isotope labeled peptides are used as internal standards. This software identifies the target peaks based on the retention time of the signal peaks of the IS peptides in each analysis. Therefore, the software can overcome the problem of different retention times of target peaks between samples and can properly automatically recognize target peaks in most chromatograms, accelerating data processing compared to conventional software.

## Discussion

### Troubleshooting

Typical problems and troubleshooting in QTAP experiments are listed in Table 8. Proteins and peptides, particularly hydrophobic ones, are likely to adsorb to the upper walls of the tubes and pipette tips and are partially lost during sample preparation, resulting in an underestimation of the protein expression levels of target proteins. Immature technique also causes irreproducible quantification results because the degrees of loss are different between samples. To improve the technique, it is important to carefully handle sample solutions to minimize the adsorption of proteins and peptides to tube walls and tips and to practice several times to smoothly execute the experiment. An artificial protein known as a “monitoring protein” is a useful tool for evaluating the proficiency of experimenters; a fixed amount of the artificial protein (A fmol) is added into every protein sample before sample preparation, the digested peptides of the artificial protein are quantified by LC-MS/MS after sample preparation (B fmol), and the recovery rate is calculated for every sample preparation procedure by determining the ratio of B/A.

**Table 8:** Typical problems and troubleshooting in QTAP

**Problem**	**Possible reasons**	**Solutions**
No signal peak is observed for authentic peptides (St or IS) in SRM/MRM analysis.	Incorrect LC conditions and/or SRM/MRM parameters (e.g., incorrect m/z of SRM/MRM transition).	Ensure that the correct conditions and parameters are used.
	Observed m/z is mismatched to theoretical m/z in MS analyzer.	Conduct mass calibration.
	Decrease in sensitivity of MS analyzer.	Clean MS device.
	The authentic solution is not injected in autosampler.	· Check the remaining volume of solution in the well. Repair if necessary.
		· Do not introduce bubbles when applying solution to wells.
	Liquid leak in LC-MS/MS system.	Determine whether there is liquid leak. Repair if necessary.
	Sensitivity of peptide is not high enough. Peptide is not retained in or not eluted from column.	Change the target peptide.
No reproducible result for quantitative values.	Loss of proteins/peptides during sample preparation due to immature technique. The extent of loss is different between samples.	· Need additional practice.
		· Add a fixed amount of artificial protein (“monitoring protein”) to every protein sample before sample preparation, and quantify the digested peptides of the artificial protein by LC-MS/MS after sample preparation to evaluate the recovery rate (%) in the sample preparation.
	The signal peak is partially occluded by background noise.	Use a high-resolution MS analyzer such as a TripleTOF5600.
	Inappropriate peak recognition or inappropriate range of calibration curve used for quantification.	· Use a basic rule of peak recognition.
		· The range of the calibration curve should be adjusted according to the expression level of the target proteins, or the sample should be diluted to be quantified within the linear range of the calibration curve.
Protein expression of target protein is not detected in any tissues or cells.	Efficacy of enzyme digestion (LysC and trypsin) is extremely low.	Change the target peptide, and avoid the transmembrane region.
	Protein expression level is below the limit of quantification in any tissues and cells.	· Use a more sensitive MS analyzer or target peptides.
		· Purify and concentrate the target protein or peptide.

Example: Comparison of protein expression levels of transporters, receptors, claudin-5, and marker proteins in brain capillaries in different mouse strains: ddY, FVB, and C57BL/6J

The availability of mouse models has facilitated significant progress in the study of CNS-related diseases because genetic engineering technologies, such as gene knockout and transgenic mice, enable the elucidation of the role of specific genes in these diseases. Several types of mouse strains are widely used in these studies, and distinct differences between the strains have been reported in behavioral and neural parameters [22]. However, thus far, there is no information regarding inter-strain differences in BBB permeability. To clarify these differences, QTAP can be used to analyze functional proteins, such as transporters, receptors, and tight junction proteins, that are involved in BBB transport. We have previously reported the absolute expression levels of transporters, receptors, claudin-5, and marker proteins in isolated brain capillaries of ddY and FVB mice [4,6,23]. However, in the present study, these were quantified again, together with the corresponding levels in C57BL/6J mice, in order to eliminate any experimental bias due to differences in experimental day and experimenter.

Table 9 shows the direct comparison of the protein expression levels in the brain capillaries of ddY, FVB, and C57BL/6J mice. A total of 13 molecules, including 7 transporters, 3 receptors, 1 tight junction protein, and 2 marker proteins, were detected among 18 molecules in all 3 mouse strains, and the differences in the protein expression levels between the 3 strains were within a 2.2-fold range for the 13 molecules. This result suggests that inter-strain differences in BBB permeability in mice are small, unlike behavioral and neural parameters.

**Table 9:** Protein expression levels of transporters, receptors, claudin-5, and marker proteins in brain capillaries isolated from ddY, FVB, and C57BL/6J mice

**Molecular name**	**Protein expression level (fmol/μg protein)**		
	**ddY**	**FVB**	**C57BL/6J**
**ABC transporters**			
Abcb1a (Mdr1a/P-gp)	16.4 ± 1.3^c^	14.2 ± 1.6^e^	17.8 ± 1.2
Abcc1 (Mrp1)	U.L.Q. (< 0.123)^c^	U.L.Q. (< 0.080)^e^	U.L.Q. (< 0.121)
Abcc4 (Mrp4)	1.33 ± 0.14^c^	1.27 ± 0.21^e^	1.51 ± 0.27
Abcc5 (Mrp5)	U.L.Q. (< 0.497)^c^	U.L.Q. (< 0.412)^e^	U.L.Q. (< 0.544)
Abcc6 (Mrp6)	U.L.Q. (< 0.478)^c^	U.L.Q. (< 0.406)^e^	U.L.Q. (< 0.312)
Abcg2 (Bcrp)	3.74 ± 0.32^c^	3.21 ± 0.49^e^	5.48 ± 0.37^a^
**SLC transporters**			
Slc2a1 (Glut1)	82.1 ± 3.0^c^	90.9 ± 3.9^e^	101 ± 4
Slc7a5 (Lat1)	2.54 ± 1.55^c^	2.11 ± 0.82^e^	1.17 ± 0.36
Slc16a1 (Mct1)	17.3 ± 1.3^c^	19.9 ± 1.0^e^	13.7 ± 0.5^b^
Slc21a2 (Pgt)	U.L.Q. (< 0.304)	U.L.Q. (< 0.277)	U.L.Q. (< 0.309)
Slc22a8 (Oat3)	1.78 ± 0.15^c^	1.65 ± 0.52^e^	2.29 ± 0.40
Slc29a4 (Pmat)	U.L.Q. (< 0.220)	U.L.Q. (< 0.194)^e^	U.L.Q. (< 0.193)
**Receptors**			
Insr	0.738 ± 0.212^d^	0.637 ± 0.080^e^	1.13 ± 0.18
Lrp1	1.36 ± 0.42^d^	0.981 ± 0.072^e^	1.37 ± 0.33
Tfr1	4.34 ± 0.81^d^	3.89 ± 0.66^e^	5.22 ± 0.47
**Tight junction protein**			
Claudin-5	6.16 ± 0.20	5.50 ± 0.49^e^	8.07 ± 1.47
**Marker proteins**			
Na^+^/K^+^-ATPase	39.5 ± 1.9^c^	32.3 ± 1.3^e^	39.0 ± 0.9
γ-gtp	3.01 ± 0.47^c^	2.45 ± 0.12^e^	3.17 ± 0.36

ddY and C57BL/6J mice were purchased from Japan SLC Inc., and FVB mice were purchased from CLEA Japan, Inc. Brain capillaries were freshly isolated from approximately 3 g of cerebrums pooled from 10 mice (adult, 10 weeks old, male) using a nylon mesh method. After the samples, including brain capillaries, were passed through 210- and 85-μm nylon mesh, the filtrate from the 85-μm nylon mesh was loaded onto the 20-μm nylon mesh. The brain capillaries retained on the 20-μm nylon mesh were used for absolute quantification. The recoveries of brain capillaries from ddY, FVB, and C57BL/6J mice were 110, 49.6 and 65.9 μg protein/g cerebrum, respectively. Whole tissue lysates of brain capillaries (50 μg protein) were digested with trypsin into peptide samples. The protein expression levels of the target proteins were measured by subjecting the peptide samples (30 or 3.33 μg protein) to LC-MS/MS with 500 fmol of stable isotope-labeled peptide mixture. The protein expression levels were calculated as an average of 6–8 quantitative values obtained from three or four SRM/MRM transitions in duplicate analyses. Each value represents the mean ± S.E.M. Briefly, this S.E.M. indicates the variability of the quantitative values between different transitions but does not indicate the variability between individual mice. ^a^*p* < 0.001, significantly different from the protein expression level in ddY and FVB mice for bcrp (Bonferroni test). ^b^*p* < 0.001, significantly different from the protein expression level in FVB mouse for mct1 (Bonferroni test). For the other molecules, there was no statistically significant difference in the protein expression levels between the three strains (*p* > 0.01, Bonferroni test). ^c, d, e^ These data were quantified again in the present study to eliminate experimental bias. The original results were reported in Kamiie et al. [4], Uchida et al. [6] and Agarwal et al. [23], respectively. U.L.Q., under limit of quantification.

The expression levels of breast cancer resistance protein (Bcrp/Abcg2) and monocarboxylate transporter 1 (Mct1/Slc16a1) were statistically significantly different between C57BL/6J mice and the other strain(s). The expression level of Bcrp/Abcg2 in C57BL/6J mice was significantly greater than that in ddY and FVB mice, whereas the Mct1 expression level in C57BL/6J mice was significantly less than that in FVB mice. At the BBB, Bcrp forms a functional barrier against drug entry into the brain by pumping drugs out of brain capillary endothelial cells. The higher level of Bcrp at the BBB of C57BL/6J mice thus implies reduced BBB drug permeability. At the BBB, Mct1 contributes to the supply of ketone bodies as an alternative source of energy to the brain, and thus the lower expression of Mct1 in C57BL/6J mice suggests a difference in brain energy metabolism compared to FVB mice.

Most of the data in ddY and FVB mice were quantified again in the present study to ensure comparability. These data were in good agreement the originally reported data [4,6,23], within ±30% in almost all cases, demonstrating the good day-to-day and experimenter-to-experimenter reproducibility of QTAP.

### Possible application and perspective

Clarifying the physiological role of the BBB and the regulation of its function are crucial for the diagnosis and prevention of CNS disease and the development of new CNS-targeted drugs. Because proteins play pivotal roles in cellular function and are the minimum unit of cellular function, it is important to clarify the absolute protein expression level in cells, the mechanism of regulation, and the modification of protein function. In this study protocol, we have introduced a detailed procedure for establishing the method, preparing the sample, and quantifying protein expression by LC-MS/MS for highly sensitive, selective, and simultaneous protein quantification. This methodology is applicable for BBB research as well as any research involving proteins. QTAP-based research is novel and will enable us to clarify several important subjects. Table 10 summarizes what were achieved by QTAP. Tables 11 and 12 summarize the potential applications of QTAP for *in vitro* and *in vivo* studies, respectively. When the assay sensitivity of MS/MS increases 10-fold compared to the current level of sensitivity and reaches 1 attomole peptide per analysis, the progress of QTAP-based research will be significantly enhanced. The quantitative analysis of modified proteins is also one of the most important subjects to clarify the mechanism of signal transduction and to identify a solution to regulate the associated mechanisms. In the future, QTAP-based studies will revolutionize the progress of BBB research.

**Table 10:** What we did with quantitative targeted absolute proteomics (QTAP)

	
1.	Protein quantification.
2.	Simultaneous quantification of hundreds of proteins in a single analysis.
3.	Peptide with a maximal sensitivity of 20 attomole/injection, which corresponds to that of antibody detection.
4.	Establishment of a quantitative assay based on only the protein sequence database; the standard protein does not need to be used to select target peptides.
5.	Optimization of the peptide sequence produced by various proteases *in silico*.
6.	Differentiation between a peptide and a peptide with a single amino acid substitution.
7.	Differentiation between a peptide and its chemically modified form.
8.	Virtual SRM/MRM-based detection of hundreds of proteins in a single preliminary assay.

**Table 11:** **What QTAP can evaluate in an *in vitro *study**

	
1.	Protein abundance in the plasma membrane and/or organelles, including the cytoplasm.
2.	Differential protein expression in the organ from which the cells originated.
3.	Differential protein expression in the different sides of membrane vesicles.
4.	Differential protein expression of cellular characteristics under various cell culture conditions such as co-culture, conditioned medium and passage number.
5.	Alteration of cellular characteristics at various stages of differentiation.
6.	Expression level of target protein(s) over-expressed in cells by transfection of the corresponding gene.
7.	Expression level of an endogenous protein(s) to select a host cell line for the transfection of an exogenous gene and to validate the up-regulation and/or down-regulation of functionally related proteins in the cells after transfection of an exogenous gene.
8.	Expression level of target proteins generated by an artificially transfected exogenous chromosome, such as one from a different species of animal, in the cells.
9.	Cellular characterization of xenograft tissues before and after their transplantation into *in vivo* animals.

**Table 12:** **What QTAP can evaluate in an *in vivo *study**

	
1.	Inter-organ difference in protein abundance.
2.	Differences in functional protein localization in various organs and their impact on pharmacokinetics, efficacy, and drug toxicity.
3.	Assay system of ADMET and efficacy based on differences in functional protein abundance in organs.
4.	Characteristics of transgenic or gene-knockout animals based on the expression levels of a target protein and other non-target proteins.
5.	Consistency between the characteristics of xenograft-transplanted animals and human diseases.
6.	Inter-colony, inter-strain, inter-sex, inter-species, inter-racial, inter-disease, and intra-disease differences in the expression levels of functional proteins.
7.	Impact of circadian rhythm and developing/aging on functional proteins.
8.	Prediction of ADMET and efficacy of drugs in animals and humans, including the diseased state, based on the absolute levels of functional proteins.
9.	Determinant factors that can affect inter-individual differences in ADMET, drug efficacy, and their impact on personalized medicine.
10.	Suitable choice of molecular target-based drugs based on the absolute levels of target proteins in a drug-targeting organ.

ADMET, absorption, distribution, metabolism, elimination, and toxicity.

## Abbreviations

AAA: Amino acid analysis; BBB: Blood–brain barrier; CE: Collision energy; CNS: Central nervous system; DP: Declustering potential; DTT: Dithiothreitol; IAA: Iodoacetamide; IS: Internal standard; LC-MS/MS: Liquid chromatography–tandem mass spectrometry; LQ: Limit of quantification; LysC: Lysyl endopeptidase C; PPx: Pharmacoproteomics; PTM: Post-translational modification; QqQ: Triple quadrupole mass spectrometer; QTAP: Quantitative targeted absolute proteomics; R.T.: Room temperature; SRM/MRM: Selected/multiple reaction monitoring; St: Standard; UHPLC: Ultra-high pressure liquid chromatography; U.L.Q.: Under limit of quantification.

## Competing interests

Tetsuya Terasaki and Sumio Ohtsuki are full professors of Tohoku University, and Kumamoto University, respectively, and are also directors of Proteomedix Frontiers Co. Ltd. Their positions at Proteomedix Frontiers do not affect the design of the study, collection of the data, analysis or interpretation of the data, decision to submit the manuscript for publication, or writing of the manuscript. The other authors declare no competing interest.

## Authors’ contributions

Conception: YU, MT, SO, and TT. Design: YU, MT, YH, YT, SO, and TT. Data acquisition: YU, YH, and YT. Analysis: YU, YH, and YT. Data interpretation: All authors. Wrote manuscript: YU, MT, WO, YH, YT, and TT. Reviewed manuscript: YU, MT, SO, and TT. Approval of final version of manuscript: All authors.

## Authors’ information

YU: Assistant professor in Graduate School of Pharmaceutical Sciences, Tohoku University, Japan.

MT: Associate professor in Graduate School of Pharmaceutical Sciences, Tohoku University, Japan.

WO, YH, and YT: Graduate students in Graduate School of Pharmaceutical Sciences, Tohoku University, Japan.

SO: Full professor in Faculty of Life Sciences, Kumamoto University, Japan.

TT: Full professor in Graduate School of Pharmaceutical Sciences, Tohoku University, Japan.

## References

[B1] Chen P Li X Sun Y Liu Z Cao R He Q Wang M Xiong J Xie J Wang X Liang S Proteomic analysis of rat hippocampal plasma membrane: characterization of potential neuronal-specific plasma membrane proteins J Neurochem 2006 98 1126 1140 10.1111/j.1471-4159.2006.03934.x 16895580

[B2] Nagano K Taoka M Yamauchi Y Itagaki C Shinkawa T Nunomura K Okamura N Takahashi N Izumi T Isobe T Large-scale identification of proteins expressed in mouse embryonic stem cells Proteomics 2005 5 1346 1361 10.1002/pmic.200400990 15742316

[B3] Pshezhetsky AV Fedjaev M Ashmarina L Mazur A Budman L Sinnett D Labuda D Beaulieu JF Menard D Nifant’ev I Levy E Subcellular proteomics of cell differentiation: quantitative analysis of the plasma membrane proteome of Caco-2 cells Proteomics 2007 7 2201 2215 10.1002/pmic.200600956 17549793

[B4] Kamiie J Ohtsuki S Iwase R Ohmine K Katsukura Y Yanai K Sekine Y Uchida Y Ito S Terasaki T Quantitative atlas of membrane transporter proteins: development and application of a highly sensitive simultaneous LC/MS/MS method combined with novel in-silico peptide selection criteria Pharm Res 2008 25 1469 1483 10.1007/s11095-008-9532-4 18219561

[B5] Ito K Uchida Y Ohtsuki S Aizawa S Kawakami H Katsukura Y Kamiie J Terasaki T Quantitative membrane protein expression at the blood–brain barrier of adult and younger cynomolgus monkeys J Pharm Sci 2011 100 3939 3950 10.1002/jps.22487 21254069

[B6] Uchida Y Ohtsuki S Katsukura Y Ikeda C Suzuki T Kamiie J Terasaki T Quantitative targeted absolute proteomics of human blood–brain barrier transporters and receptors J Neurochem 2011 117 333 345 10.1111/j.1471-4159.2011.07208.x 21291474

[B7] Ohtsuki S Ikeda C Uchida Y Sakamoto Y Miller F Glacial F Decleves X Scherrmann JM Couraud PO Kubo Y Tachikawa M Terasaki T Quantitative targeted absolute proteomic analysis of transporters, receptors and junction proteins for validation of human cerebral microvascular endothelial cell line hCMEC/D3 as a human blood–brain barrier model Mol Pharm 2013 10 289 296 10.1021/mp3004308 23137377

[B8] Ohtsuki S Uchida Y Kubo Y Terasaki T Quantitative targeted absolute proteomics-based ADME research as a new path to drug discovery and development: methodology, advantages, strategy, and prospects J Pharm Sci 2011 100 3547 3559 10.1002/jps.22612 21560129

[B9] Uchida Y Ohtsuki S Kamiie J Terasaki T Blood–brain barrier (BBB) pharmacoproteomics: reconstruction of in vivo brain distribution of 11 P-glycoprotein substrates based on the BBB transporter protein concentration, in vitro intrinsic transport activity, and unbound fraction in plasma and brain in mice J Pharmacol Exp Ther 2011 339 579 588 10.1124/jpet.111.184200 21828264

[B10] Anderson L Hunter CL Quantitative mass spectrometric multiple reaction monitoring assays for major plasma proteins Mol Cell Proteomics 2006 5 573 588 16332733 10.1074/mcp.M500331-MCP200

[B11] Kawakami H Ohtsuki S Kamiie J Suzuki T Abe T Terasaki T Simultaneous absolute quantification of 11 cytochrome P450 isoforms in human liver microsomes by liquid chromatography tandem mass spectrometry with In silico target peptide selection J Pharm Sci 2011 100 341 352 10.1002/jps.22255 20564338

[B12] Niessen J Jedlitschky G Grube M Bien S Schwertz H Ohtsuki S Kawakami H Kamiie J Oswald S Starke K Strobel U Siegmund W Rosskopf D Greinacher A Terasaki T Kroemer HK Human platelets express organic anion-transporting peptide 2B1, an uptake transporter for atorvastatin Drug Metab Dispos 2009 37 1129 1137 10.1124/dmd.108.024570 19237515

[B13] Niessen J Jedlitschky G Grube M Kawakami H Kamiie J Ohtsuki S Schwertz H Bien S Starke K Ritter C Strobel U Greinacher A Terasaki T Kroemer HK Expression of ABC-type transport proteins in human platelets Pharmacogenet Genomics 2010 20 396 400 10.1097/FPC.0b013e32833997b0 20395880

[B14] Obuchi W Ohtsuki S Uchida Y Ohmine K Yamori T Terasaki T Identification of transporters associated with Etoposide sensitivity of stomach cancer cell lines and methotrexate sensitivity of breast cancer cell lines by quantitative targeted absolute proteomics Mol Pharmacol 2013 83 490 500 10.1124/mol.112.081083 23197647

[B15] Ohmine K Kawaguchi K Ohtsuki S Motoi F Egawa S Unno M Terasaki T Attenuation of phosphorylation by deoxycytidine kinase is key to acquired gemcitabine resistance in a pancreatic cancer cell line: targeted proteomic and metabolomic analyses in PK9 cells Pharm Res 2012 29 2006 2016 10.1007/s11095-012-0728-2 22419259

[B16] Ohtsuki S Schaefer O Kawakami H Inoue T Liehner S Saito A Ishiguro N Kishimoto W Ludwig-Schwellinger E Ebner T Terasaki T Simultaneous absolute protein quantification of transporters, cytochromes P450, and UDP-glucuronosyltransferases as a novel approach for the characterization of individual human liver: comparison with mRNA levels and activities Drug Metab Dispos 2012 40 83 92 10.1124/dmd.111.042259 21994437

[B17] Yoneyama T Ohtsuki S Ono M Ohmine K Uchida Y Yamada T Tachikawa M Terasaki T Quantitative targeted absolute proteomics-based large-scale quantification of proline-hydroxylated alpha-fibrinogen in plasma for pancreatic cancer diagnosis J Proteome Res 2013 12 753 762 10.1021/pr3008144 23298340

[B18] Yoshikawa A Nakada M Ohtsuki S Hayashi Y Obuchi W Sato Y Ikeda C Watanabe T Kawahara Y Hasegawa T Sabit H Kita D Nakanuma Y Terasaki T Hamada JI Recurrent anaplastic meningioma treated by sunitinib based on results from quantitative proteomics Neuropathol Appl Neurobiol 2012 38 105 110 10.1111/j.1365-2990.2011.01197.x 21696419

[B19] Shawahna R Uchida Y Decleves X Ohtsuki S Yousif S Dauchy S Jacob A Chassoux F Daumas-Duport C Couraud PO Terasaki T Scherrmann JM Transcriptomic and quantitative proteomic analysis of transporters and drug metabolizing enzymes in freshly isolated human brain microvessels Mol Pharm 2011 8 1332 1341 10.1021/mp200129p 21707071

[B20] Ohtsuki S Yamaguchi H Asashima T Terasaki T Establishing a method to isolate rat brain capillary endothelial cells by magnetic cell sorting and dominant mRNA expression of multidrug resistance-associated protein 1 and 4 in highly purified rat brain capillary endothelial cells Pharm Res 2007 24 688 694 10.1007/s11095-006-9188-x 17318419

[B21] Pardridge WM Yang J Eisenberg J Tourtellotte WW Isolation of intact capillaries and capillary plasma membranes from frozen human brain J Neurosci Res 1987 18 352 357 10.1002/jnr.490180213 3694717

[B22] Ingram DK Jucker M Developing mouse models of aging: a consideration of strain differences in age-related behavioral and neural parameters Neurobiol Aging 1999 20 137 145 10.1016/S0197-4580(99)00033-0 10537023

[B23] Agarwal S Uchida Y Mittapalli RK Sane R Terasaki T Elmquist WF Quantitative proteomics of transporter expression in brain capillary endothelial cells isolated from P-glycoprotein (P-gp), breast cancer resistance protein (Bcrp), and P-gp/Bcrp knockout mice Drug Metab Dispos 2012 40 1164 1169 10.1124/dmd.112.044719 22401960 PMC3362790

